# TB and HIV among hill tribe marginalized vulnerable population, Thailand

**DOI:** 10.1186/1742-4690-9-S1-P88

**Published:** 2012-05-25

**Authors:** Tawatchai Apidechkul

**Affiliations:** 1 School of Health Science, Mae Fah Luang University, Chiang Rai Province, Thailand

## Introduction

Since 1982, Thailand had been reported 372,874 cases of HIV/AIDS, and 98,153 deaths. The north of Thailand has been report as the highest prevalence areas. There were almost 600,000 hill tribe populations live there as a marginalized and vulnerable people under lacked of access to health care and limited education. Most of them emigrated from China last 200 years ago. Chiang Rai Province is the most favorite living area of hill tribe people.

## Materials and methods

The retrospective cohort study design aimed to investigates the TB and HIV situation among hill tribe marginalized and vulnerable population. The systematic data collection with the completed questionnaire was conducted in the 12 hospitals, Chiang Rai Province. All questionnaires had been tested for reliability and validity before use. Survival and Cox’s regression were analysis.

## Results

Of 629 cases of TB reported during 2009-2011 form 12 hospitals were recruited into the study. 60.7% were male 23.8% aged 51-60 years old, and followed by 41-50 years old (20.2%) (min=1, max=93). Of 84.6%were pulmonary TB and extra pulmonary 15.4%, 44.6%recieving AFB testing, 77.4%new cases. The results of treatment found that 22.7% were cure, 28.6%complte, 4.3%defults, 8.1%death, and 1.9% failure. Prevalence of HIV/AIDS among hill tribe TB cases was 17.2%. Of 88.1% had treatment on CAT1, and 4.6%CAT2, and 2.3%CAT4. Male had greater pulmonary TB (p-vale=0.044), and HIV+ than female (p-value=0.023). Survival analysis found that being male (p-value=0.01), non-HIV (p-value<0.01), and CAT1 had greater success treatment. Cox’s regression found that only aged 11-20 years old had related to success treatment (HR=2.11, 95%CI=1.05-4.26).

## Conclusion

Active screening program and increasing the rights of access to care are immediate needed for hill tribe vulnerable people for coping TB problem in Thailand.

